# Effects of dietary *Lactobacillus* postbiotics and bacitracin on the modulation of mucosa-associated microbiota and pattern recognition receptors affecting immunocompetence of jejunal mucosa in pigs challenged with enterotoxigenic F18^+^ *Escherichia coli*

**DOI:** 10.1186/s40104-024-01098-1

**Published:** 2024-10-11

**Authors:** Marcos Elias Duarte, Zixiao Deng, Sung Woo Kim

**Affiliations:** Department of Animal Science, North Carolina State University, 116 Polk Hall, Campus Box 7621, Raleigh, NC 27695 USA

**Keywords:** *Escherichia coli*, Immunocompetence, Intestinal health, Pattern recognition receptors, Pigs

## Abstract

**Background:**

Enterotoxigenic *Escherichia coli* (*E. coli*) is a threat to humans and animals that causes intestinal disorders. Antimicrobial resistance has urged alternatives, including *Lactobacillus* postbiotics, to mitigate the effects of enterotoxigenic *E. coli*.

**Methods:**

Forty-eight newly weaned pigs were allotted to NC: no challenge/no supplement; PC: F18^+^ *E. coli* challenge/no supplement; ATB: F18^+^ *E. coli* challenge/bacitracin; and LPB: F18^+^ *E. coli* challenge/postbiotics and fed diets for 28 d. On d 7, pigs were orally inoculated with F18^+^ *E. coli*. At d 28, the mucosa-associated microbiota, immune and oxidative stress status, intestinal morphology, the gene expression of pattern recognition receptors (PRR), and intestinal barrier function were measured. Data were analyzed using the MIXED procedure in SAS 9.4.

**Results:**

PC increased (*P* < 0.05) *Helicobacter mastomyrinus* whereas reduced (*P* < 0.05) *Prevotella copri* and *P. stercorea* compared to NC. The LPB increased (*P* < 0.05) *P. stercorea* and *Dialister succinatiphilus* compared with PC. The ATB increased (*P* < 0.05) *Propionibacterium acnes*, *Corynebacterium glutamicum*, and *Sphingomonas pseudosanguinis* compared to PC. The PC tended to reduce (*P* = 0.054) *PGLYRP4* and increased (*P* < 0.05) *TLR4*, *CD14*, MDA, and crypt cell proliferation compared with NC. The ATB reduced (*P* < 0.05) *NOD1* compared with PC. The LPB increased (*P* < 0.05) *PGLYRP4*, and interferon-γ and reduced (*P* < 0.05) *NOD1* compared with PC. The ATB and LPB reduced (*P* < 0.05) TNF-α and MDA compared with PC.

**Conclusions:**

The F18^+^ *E. coli* challenge compromised intestinal health. Bacitracin increased beneficial bacteria showing a trend towards increasing the intestinal barrier function, possibly by reducing the expression of PRR genes. *Lactobacillus* postbiotics enhanced the immunocompetence of nursery pigs by increasing the expression of interferon-γ and PGLYRP4, and by reducing TLR4, NOD1, and CD14.

## Background

The improved immunocompetency of intestinal mucosa is crucial for the overall well-being and productivity of animals [1, 2]. The intestine, specifically the mucosa, plays a pivotal role in nutrient absorption, immunological defense, and establishing a symbiotic relationship with the intestinal microbiota [3, 4]. Disruptions in the balance of the intestinal microbiota and mucosal integrity can lead to dysbiosis, impaired immune function, and increased susceptibility to enteric pathogens [5]. Enterotoxigenic *Escherichia coli* (*E. coli*) is a significant pathogen responsible for causing enteric diseases in both humans and animals [6–8]. In pigs, F18^+^ *E. coli* infections lead to inflammation, oxidative damages, and villus destruction in the jejunum, resulting in post-weaning diarrhea, and decreased growth [8].

Traditional approaches to combat enterotoxigenic *E. coli* infections have relied heavily on the use of antibiotics [8]. However, the overuse and misuse of antibiotics have contributed to the emergence of antibiotic-resistant strains, posing a serious threat to both human and animal health [9, 10]. Therefore, alternative strategies are urgently needed to mitigate the impact of enterotoxigenic *E. coli* infections without exacerbating the antibiotic resistance crisis [9, 11].

One potential alternative is the use of postbiotics derived from microorganisms that confer health benefits to the host [12–15]. Lactobacilli is a well-known group of bacteria that has been extensively studied for its potential to modulate the intestinal microbiota, strengthen the intestinal barrier, and regulate the immune response [15–17]. *Lactobacillus* postbiotics have shown promise in improving intestinal health and reducing the severity of various gastrointestinal disorders in both humans and animals [12, 15, 17–19]. The health benefits promoted by postbiotics can be associated with the non-living microbial cells, and their components, as well as the metabolites produced during fermentation [14]. Molecules in the microbial cell wall and metabolites interact with the host by pattern recognition receptors (PRR), modulating the host immune responses [20, 21]. The cell wall of *Lactobacillus* contains peptidoglycans that is known to modulate immune responses in the intestinal mucosa [22, 23].

The host immune system can also, modulate the microbiome within the intestine [21, 24]. The cross-talk between the immune system and the intestinal microbiota plays an essential role in immunocompetence of the host [3, 21]. Furthermore, mucosa-associated microbiota interacts more directly with the immune system compared to the luminal microbiota [25, 26]. Although the luminal microbiota differs from the mucosa-associated microbiota in composition and functions, they are not completely unrelated, and dietary interventions can modulate the microbiota in both the lumen and mucosa [27].

Therefore, it was hypothesized that dietary *Lactobacillus* postbiotics increases the immunocompetence of pigs by modulating the mucosa-associated microbiota and the PRR, reducing mucosal damage and inflammation in pigs challenged with F18^+^ *E. coli*. This study aimed to evaluate the role of dietary *Lactobacillus* postbiotics on the modulation of mucosa-associated microbiota and PRR, and its impacts on mucosal immune response in pigs challenged with F18^+^ *E. coli.*

## Materials and methods

This study followed the experimental protocol revised and approved by the Institutional Animal Care and Use Committee of North Carolina State University (Raleigh, NC, USA).

### Animals, experimental design, and diets

Forty-eight pigs (24 barrows and 24 gilts) with 7.9 ± 0.5 kg of body weight (BW) were randomly assigned to four treatments. Pigs were blocked based on sex (barrows and gilts) and initial BW (lighter and heavier) and allotted based on a randomized complete block design. The treatments were NC: no challenge/no supplement; PC: F18^+^ *E. coli* challenge/no supplement; ATB: F18^+^ *E. coli* challenge/antibiotic (bacitracin 30 g/t feed); and LPB: F18^+^ *E. coli* challenge/*Lactobacillus* postbiotics (2 kg/t feed). Bacitracin methylene disalicylate was used as a source of bacitracin. The postbiotics contained 6 × 10^10^/g of powder of heat-inactivated *Lactobacillus* (*L. fermentum* and *L. delbrueckii*) as well as the spent media (LBiotix, Adare Biome, Houdan, France). The dosage was set based on previous study conducted by Xu et al. [12] and Warda et al. [17]. Pigs were fed a basal diet formulated to meet the nutrient requirements of NRC (2012) [28], as shown in Table 1. The ATB and LPB were added to the basal diet at the expense of corn.

**Table 1:** Composition of experimental diet

Feedstuff	Basal diet
Corn	54.62
Soybean meal	23.50
Whey permeate	10.00
Poultry meal	4.00
Blood plasma	3.00
Poultry fat	2.00
L-Lysine HCl	0.47
L-Methionine	0.18
L-Threonine	0.13
Dicalcium phosphate	0.85
Limestone	0.85
Vitamin premix^a^	0.03
Mineral premix^b^	0.15
Salt	0.22
Calculated nutrient composition	
ME, kcal/kg	3,403
Crude protein, %	21.55
SID^c^ Lys, %	1.35
SID Met + Cys, %	0.74
SID Trp, %	0.22
SID Thr, %	0.79
SID Val, %	0.86
Ca, %	0.80
STTD^d^ P, %	0.40

^a^The vitamin premix provided the following per kilogram of complete diet: 6,613.8 IU of vitamin A as vitamin A acetate, 992.0 IU of vitamin D_3_, 19.8 IU of vitamin E, 2.64 mg of vitamin K as menadione sodium bisulfate, 0.03 mg of vitamin B_12_, 4.63 mg of riboflavin, 18.52 mg of D-pantothenic acid as calcium pantothenate, 24.96 mg of niacin, and 0.07 mg of biotin

^b^The trace mineral premix provided the following per kilogram of complete diet: 4.0 mg of Mn as manganous oxide, 165 mg of Fe as ferrous sulfate, 165 mg of Zn as zinc sulfate, 16.5 mg of Cu as copper sulfate, 0.30 mg of I as ethylenediamine dihydroiodide, and 0.30 mg of Se as sodium selenite

^c^SID: standardized ileal digestible

^d^STTD: standardized total tract digestible

The strain F18ac (O147) that produces heat-stable toxin A (STa) and heat-stable toxin B (STb) was used for this study. The F18ac (O147) was selected based on its strong capacity to adhere to the small intestinal receptors in newly weaned pigs [29]. The dosage of inoculation was 2.4 × 10^10^ CFU and the culture of F18^+^ *E. coli* was prepared following the protocol as previously reported [12, 30].

### Sample collection

Following the 28 d feeding period, the pigs were euthanized by exsanguination subsequent to a penetrating captive bolt applied to the head. Immediately after euthanasia, a section of 15 cm from mid-jejunum (3 m after the duodenojejunal junction) was rinsed with sterile saline solution (0.9% NaCl) and opened for collection of samples. The mucosa samples were obtained by gently scraping the jejunal section with a microscope slide. Subsequently, the samples were placed in 2-mL tubes (two sets per pig) and rapidly frozen using liquid nitrogen before being stored in a freezer at −80 °C for analysis of mucosa-associated microbiota, immune and oxidative stress markers. Jejunal tissue (2 cm) was collected and stored at −80 °C (after snap-freezing in liquid nitrogen, immediately after collection) for further measurements of the gene expression of intestinal biomarkers associated with microbial cell wall components and intestinal barrier function. Another jejunal section (5 cm) was fixed in 10% formalin to be used for histological evaluation.

### Mucosa-associated microbiota

The DNA was extracted from the jejunal mucosa samples to analyze the mucosa-associated microbiota using the 16S rRNA sequencing as described by Duarte et al. [31]. The extraction process utilized the DNA Stool Mini Kit (#51604, Qiagen; Germantown, Maryland, USA) and followed the manufacturer’s instructions. The DNA samples were then sent to Mako Medical Laboratories (Raleigh, NC, USA) for microbial sequencing. To prepare the samples for sequencing, the Ion Chef instrument was employed, and the sequencing was conducted on the Ion S5 system (Thermo Fisher, Wilmington, DE, USA). The amplification of variable regions V2, V3, V4, V6, V7, V8, and V9 of the 16S rRNA gene was accomplished using the Ion 16S Metagenomics Kit 113 (Thermo Fisher Scientific). The obtained sequences were then processed using the Torrent Suite Software (version 5.2.2) (Thermo Fisher Scientific), resulting in raw unaligned sequence data files for subsequent analysis. For the analysis of the sequence data, including alignment to the GreenGenes and MicroSeq databases, generation of alpha and beta diversity plots, and creation of an operational taxonomic unit (OTU) table, the Ion Reporter Software Suite (version 5.2.2) of bioinformatics analysis tools (Thermo Fisher Scientific) was employed. The Ion Reporter’s Metagenomics 16S workflow powered by Qiime (version w1.1) was utilized to analyze the samples. Before statistical analysis, the OTU data were converted to relative abundance. The OTU with a relative abundance of less than 0.05% at each level were combined and labeled as “Others” as previously reported by Deng et al. [32]. The microbial diversity was evaluated by alpha-diversity (Chao1, Shannon, and Simpson) and beta-diversity (Bray–Curtis distance) following Deng et al. [33].

### Biomarkers associated with microbial cell wall

The mid-jejunal tissue was used to extract the total RNA using TRIzol reagent (Thermo Fisher Scientific) as previously described by Duarte and Kim [34]. The concentration of total RNA was measured using a nano-volume spectrophotometer. The complementary DNA was synthesized using 1 µg of total RNA and the oligo dT and M-MLV Reverse Transcriptase (Thermo Fisher Scientific) according to the manufacturer’s instructions. Relative levels of messenger ribonucleic acid (mRNA) were measured by quantitative real-time polymerase chain reaction (PCR) using Applied Biosystems SYBR Green PCR Master Mix (Thermo Fisher Scientific) and a QS5 Real-Time PCR System. The Ct of housekeeping gene β-actin or Villin did not differ among dietary treatments. The relative expression of each gene was normalized to β-actine or Villin using the delta–delta–Ct method as described previously [35] and expressed as the level relative to β-actine or Villin. All primers (Table 2) were verified for melting curve, efficiency (100% ± 10%), and linearity (*r*^2^ ≥ 0.99) of amplification.

**Table 2:** Sequence of primers for intestinal markers in the jejunum

Gene	Prime	Sequence (5′→3′)	Accession number	Size, bp
*PGLYRP1*	Forward	GCAAACTGCATCCCCATTGT	NM_001001260	212
	Reverse	AGGAAGTTGTAGCCCACGTC		
*PGLYRP2A*	Forward	CCAGGAACAGGTATGGGGGAC	AF541955	128
	Reverse	CCTCAGTGAACTCCTTGGCG		
*PGLYRP2B*	Forward	TGGTAAACCTGCCCTTGGAC	NM_213738.1	319
	Reverse	AAGTGTAGGCCCAGGTCTCT		
*PGLYRP3*	Forward	TCTCATGGCCCATACGCAAG	NM_001244361.1	273
	Reverse	CGACACCCTCGTACACTCTG		
*PGLYRP4*	Forward	AGTGTCACAACCAGACCAGG	NM_213737.1	195
	Reverse	AACCTGATACAACCACAACCCA		
*TLR2*	Forward	GGGCTGCGTTCATTCATCAG	XM_005653576.3	132
	Reverse	CTGCAGAGGATGGATGGCAA		
*TLR4*	Forward	CGTGCAGGTGGTTCCTAACA	NM_001113039.2	326
	Reverse	GGTTTGTCTCAACGGCAACC		
*NOD1*	Forward	AACACCGATCCAGTGAGCAG	NM_001114277.1	230
	Reverse	AAATGGTCTCGCCCTCCTTG		
*NOD2*	Forward	GTGCCTCCCCTCTAGACTCA	NM_001105295.1	191
	Reverse	ACGAACCAGGAAGCCAAGAG		
*CD14*	Forward	CCCTGCCAAATAGACGACGA	NM_001097445.2	299
	Reverse	TCGAGCGTCAGTTCCTTGAG		
*CD3*	Forward	GTGGATCTGATGGCAGTGGT	NM_214227.1	205
	Reverse	TCCGGATGGGCTCATAGTCT		
*IFN-γ*	Forward	GGCCATTCAAAGGAGCATGG	HQ026021.1	119
	Reverse	AAGCTCATCTCACCGGAATTT		
*NF-κB*	Forward	GCTGGAATGAAGCACGGAAC	NM_001048232.1	236
	Reverse	GCAAGTTGCATGGCCTTCTC		
*ZO-1*	Forward	CAGAGACCAAGAGCCGTCC	XM_003480423.4	105
	Reverse	TGCTTCAAGACATGGTTGGC		
Claudin 1	Forward	AAACCGTGTGGGAACAACCA	NM_001244539.1	196
	Reverse	CACATGAAAATGGCTTCCCTC		
Occludin	Forward	CAGGCTGCGGTGAGAAGATT	XP_005672579.1	169
	Reverse	TATGTCGTTGCTGGGTGCAT		
*MUC2*	Forward	CAACGGCCTCTCCTTCTCTGT	XM_021082584.1	70
	Reverse	GCCACACTGGCCCTTTGT		
β-actin	Forward	CAAATGCTTCTAGGCGGACTGT	XM_003124280.5	75
	Reverse	TCTCATTTTCTGCGCAAGTTAGG		
Villin	Forward	ACGTGTCTGACTCCGAGGGAAAGGT	XM_001925167.6	201
	Reverse	ACTGCTTCGCTTTGATAAAGTTCAG		

### Immune parameters and oxidative stress

Before assays, 1 g of the jejunal mucosa samples was mixed with 1 mL of phosphate-buffered saline (PBS) and homogenized on ice using a tissue homogenizer. The mixture was subjected to centrifugation at 13,000 × *g* for 15 min. The resulting supernatant was carefully collected and divided into five sets, each containing 150 µL of the sample, and subsequently stored at −80 °C to maintain their integrity for subsequent analysis.

The concentrations of protein, tumor necrosis factor-alpha (TNF-α), interleukin 8 (IL-8), malondialdehyde (MDA), and protein carbonyl were determined using colorimetric methods. Commercially available assay kits were employed, following the instructions provided by the manufacturers. Absorbance readings were obtained using an ELISA plate reader (Synergy HT, BioTek Instruments, Winooski, VT, USA) and analyzed with Gen5 Data Analysis Software (BioTek Instruments). The concentrations were calculated based on the standard curve generated from the known concentrations and corresponding absorbance values of the standards.

The concentration of total protein in the jejunum mucosa was determined using the BCA (bicinchoninic acid) Protein Assay (23225#, Thermo Fisher Scientific) following the protocol described by Cheng et al. [36]. Before analysis, the samples were diluted (1:60) in PBS to ensure they fell within the working range of 0 to 2,000 µg/mL. The absorbance was then measured at 562 nm. The obtained total protein concentration was utilized to normalize the concentrations of TNFα, IL-8, MDA, and protein carbonyl.

The concentration of TNF-α in the mucosa was measured using the Porcine TNF-alpha Quantikine ELISA Kit (PTA00; R&D System Inc., Minneapolis, MN, USA), following the method described by Deng et al. [32]. The standard was used within a working range of 0 to 1,500 pg/mL. Absorbance readings were taken at 450 and 550 nm, and the TNF-a concentration in the mucosa was expressed as pg/mg protein. The concentration of IL-8 in the mucosa was measured using the Porcine IL-8/CXCL8 Quantikine ELISA Kit (P8000; R&D System), following the method described by Jang et al. [37]. The standard was used within a working range of 0 to 2,000 pg/mL. Absorbance readings were taken at 450 and 550 nm, and the IL-8 concentration in the mucosa was expressed as ng/mg protein.

For the measurement of MDA concentrations in the mucosa, the OxiSelect TBARS Assay Kit (STA-330, Cell Biolabs, San Diego, CA, USA) was utilized. The MDA standard was used within a working range of 0 to 125 mmol/L. The absorbance at 532 nm was measured, and the MDA concentration was expressed as nmol/mg protein. The concentration of protein carbonyl was determined using the OxiSelect Protein Carbonyl ELISA Kit (STA-310, Cell Biolabs, CA, USA). Before measurement, mucosa samples were diluted in PBS to achieve a protein concentration of 10 µg/mL. The standard was prepared by combining oxidized BSA and reduced BSA, resulting in a working range of 0 to 7.5 nmol/mg protein. The absorbance at 450 nm was measured, and the protein carbonyl concentration was expressed as nmol/mg protein for both serum and mucosa.

### Jejunal morphology and crypt cell proliferation

Mid-jejunal tissues were used to determine intestinal morphology and crypt cell proliferation. The samples were initially fixed in 10% buffered formaldehyde for 48 h. Two sections, approximately 2 mm each, were cut from the fixed tissue, placed in a cassette, and transferred to a 70% ethanol solution. Subsequently, the samples were sent to the Histology Laboratory at the University of North Carolina (UNC School of Medicine, Chapel Hill, NC, USA) for dehydration, embedding, and Ki-67 staining. Automated Ki-67 staining was performed using the Biocare Intellipath Stainer (Biocare Medical, Pacheco, CA, USA). The primary monoclonal antibody for Ki-67 (#ACR325, Biocare Medical) was diluted 1:100 and incubated with the slides at room temperature for 30 min. Detection was done by utilizing Vector ImmPress Rabbit polymer, with staining processed by using the chromogen diaminobenzidine (DAB).

Intestinal morphology, including villus height, villus width, and crypt depth, was assessed at 40× magnification using an Olympus CX31 microscope (Lumenera Corporation, Ottawa, Canada) and Infinity 2–2 digital CCD software. Ten representative villi and crypts were selected from each pig for analysis. Villus height (VH) was measured from the tip to the junction with the crypt, while villus width was recorded at the midpoint. Crypt depth (CD) was measured from the base of the villus to the bottom of the crypt [38]. The villus height to crypt depth ratio (VH:CD) was calculated by dividing VH by CD. The percentage of Ki-67 positive cells, indicating cell proliferation within the crypts, was determined from images of 10 complete crypts captured at 100× magnification [39]. All morphological analyses were performed by the same individual, with the average of 10 measurements per sample reported as a single value.

### Growth performance and fecal score

Growth performance was evaluated by measuring average daily gain (ADG), average daily feed intake (ADFI), and the gain to feed ratio (G:F) on d 0, 7, 14, 21, and 28. Health status was assessed through fecal scores recorded throughout the study. Daily fecal scores for each pig were averaged weekly (d 0 to 7, d 7 to 14, d 14 to 21, and d 21 to 28) before statistical analysis. The fecal score was based on 1 to 5 scale [40] as follows: (1) very hard and dry stool, (2) firm stool, (3) normal stool, (4) loose stool, and (5) watery stool with no shape.

### Statistical analysis

Data were analyzed using the Proc mixed of SAS 9.4 software (SAS Inc., Cary, NC, USA). Dietary treatments were defined as fixed effects and the random effects were blocks. The experimental unit was the pig, individually housed and fed. The analysis of similarities (ANOSIM) was performed to evaluate the beta diversity of mucosa-associated microbiota. The data were visualized using principal coordinates analysis (PCA) based on Bray-Curtis distance. Statistical differences were considered significant with *P* < 0.05 and tendency with 0.05 ≤ *P* < 0.10.

## Results

### Mucosa-associated microbiota

The relative abundance of mucosa-associated microbiota at the phylum level in pigs on LPB did not differ from pigs on PC (Table 3). The relative abundances of Firmicutes and Bacteroidetes in the jejunal mucosa of pigs on PC were lower (*P* < 0.05) than in pigs on NC. The relative abundance of Proteobacteria in the jejunal mucosa of pigs on PC was greater (*P* < 0.05) than in pigs on NC. The relative abundance of Actinobacteria in the jejunal mucosa of pigs on PC was lower (*P* < 0.05) than in pigs on ATB.

**Table 3:** Relative abundance of the jejunal mucosa-associated microbiota at the phylum level in pigs challenged with F18^+^ *Escherichia coli* (*E. coli*) and fed diets supplemented with bacitracin or *Lactobacillus* postbiotics

Item	Treatment^a^				SEM	*P*-value		
	NC	PC	ATB	LPB		NC vs. PC	PC vs. ATB	PC vs. LPB
Firmicutes	39.65	20.28	22.82	22.44	5.83	0.009	0.722	0.763
Bacteroidetes	30.97	13.19	12.44	27.34	6.61	0.047	0.933	0.112
Proteobacteria	27.90	63.38	56.75	47.20	8.04	0.001	0.504	0.107
Actinobacteria	0.69	1.91	6.53	2.43	1.08	0.427	0.004	0.734
Others	0.79	1.24	1.46	0.58	0.57	0.543	0.773	0.375

^a^Treatments: NC, no challenge/no supplement; PC, F18^+^ *E. coli* challenge/no supplement; ATB, F18^+^ *E. coli* challenge/bacitracin (30 g/t feed); and LPB, F18^+^ *E. coli* challenge/*Lactobacillus* postbiotics (2 kg/t feed; LBiotix, Adare Biome, Houdan, France)

The relative abundance of Helicobacteraceae in the jejunal mucosa of pigs on PC was greater (*P* < 0.05) than in pigs on NC (Table 4). The relative abundance of Prevotellaceae and Lactobacillaceae in the jejunal mucosa of pigs on PC was lower (*P* < 0.05) than in pigs on NC. The relative abundance of Sphingomonadaceae, Propionibacteriaceae, Comamonadaceae, and Corynebacteriaceae in the jejunal mucosa of pigs on PC was lower (*P* < 0.05) than in pigs on ATB. The relative abundance of Pseudomonadaceae, Campylobacteraceae, and Others in the jejunal mucosa of pigs on PC tended to be lower (*P* = 0.052; 0.087; 0.083, respectively) than in pigs on ATB. The relative abundance of Lachnospiraceae in the jejunal mucosa of pigs on PC tended to be lower than in pigs on NC (*P* = 0.062) and LPB (*P* = 0.097). The relative abundance of Campylobacteraceae in the jejunal mucosa of pigs on PC tended to be lower than in pigs on NC (*P* = 0.062) and ATB (*P* = 0.087).

**Table 4:** Relative abundance of the jejunal mucosa-associated microbiota at the family level in pigs challenged with F18^+^ *Escherichia coli* (*E. coli*) and fed diets supplemented with bacitracin or *Lactobacillus* postbiotics

Item	Treatment^a^				SEM	*P*-value		
	NC	PC	ATB	LPB		NC vs. PC	PC vs. ATB	PC vs. LPB
Helicobacteraceae	22.05	53.15	36.67	37.62	8.60	0.004	0.112	0.134
Prevotellaceae	30.63	12.65	11.97	26.57	8.67	0.043	0.938	0.114
Lactobacillaceae	25.03	11.06	7.89	7.09	6.74	0.043	0.640	0.559
Veillonellaceae	4.56	3.34	3.64	5.35	1.04	0.372	0.823	0.143
Streptococcaceae	5.02	2.89	4.69	4.62	1.15	0.196	0.274	0.293
Sphingomonadaceae	0.44	2.21	6.80	1.64	1.42	0.267	0.005	0.720
Succinivibrionaceae	1.62	2.01	1.19	1.14	0.73	0.705	0.425	0.395
Pseudomonadaceae	1.14	0.86	3.18	0.58	0.82	0.813	0.052	0.808
Lachnospiraceae	2.11	0.80	0.52	1.96	0.50	0.071	0.694	0.108
Methylobacteriaceae	0.17	0.93	2.70	1.13	0.89	0.512	0.127	0.861
Clostridiaceae	1.43	0.96	1.90	1.23	0.56	0.555	0.237	0.736
Propionibacteriaceae	0.12	0.62	3.36	0.76	0.68	0.603	0.006	0.889
Comamonadaceae	0.16	1.03	2.75	0.81	0.58	0.287	0.039	0.786
Corynebacteriaceae	0.20	0.50	2.34	0.72	0.58	0.709	0.026	0.784
Pasteurellaceae	0.05	0.23	0.27	2.53	1.09	0.909	0.980	0.143
Campylobacteraceae	1.10	0.18	1.02	0.57	0.34	0.062	0.087	0.426
Moraxellaceae	0.30	1.60	0.64	0.31	0.56	0.106	0.231	0.108
Others	3.87	4.96	8.46	5.37	1.40	0.584	0.083	0.838

^a^Treatments: NC, no challenge/no supplement; PC, F18^+^ *E. coli* challenge/no supplement; ATB, F18^+^ *E. coli* challenge/bacitracin (30 g/t feed); and LPB, F18^+^ *E. coli* challenge/*Lactobacillus* postbiotics (2 kg/t feed; LBiotix, Adare Biome, Houdan, France)

The relative abundance of *Helicobacter mastomyrinus* in the jejunal mucosa of pigs on PC was greater (*P* < 0.05) than in pigs on NC and tended to be greater (*P* = 0.078) than in pigs on ATB (Table 5). The relative abundance of *Prevotella copri* and *Prevotella stercorea* in the jejunal mucosa of pigs on PC was lower (*P* < 0.05) than in pigs on NC, whereas the relative abundance of *Prevotella stercorea* in the jejunal mucosa of pigs on PC was lower (*P* < 0.05) than in pigs on LPB. The relative abundance of *Pelomonas puraquae* in the jejunal mucosa of pigs on PC tended to be lower (*P* = 0.070) than in pigs on ATB. The relative abundance of *Lactobacillus delbrueckii* in the jejunal mucosa of pigs on PC tended to be greater than in pigs on ATB (*P* = 0.065) and LPB (*P* = 0.085). The relative abundance of *Propionibacterium acnes*, *Corynebacterium glutamicum*, and *Sphingomonas pseudosanguinis* in the jejunal mucosa of pigs on PC was lower (*P* < 0.05) than in pigs on ATB. The relative abundance of *Roseburia faecis* in the jejunal mucosa of pigs on PC tended to be lower (*P* = 0.050) than in pigs on NC. The relative abundance of *Dialister succinatiphilus* in the jejunal mucosa of pigs on PC was lower (*P* < 0.05) than in pigs on LPB.

**Table 5:** Relative abundance of the jejunal mucosa-associated microbiota at the species level in pigs challenged with F18^+^ *Escherichia coli* (*E. coli*) and fed diets supplemented with bacitracin or *Lactobacillus* postbiotics

Item	Treatment^a^				SEM	*P*-value		
	NC	PC	ATB	LPB		NC vs. PC	PC vs. ATB	PC vs. LPB
*Helicobacter mastomyrinus*	7.83	30.76	15.86	26.18	6.63	0.009	0.082	0.587
*Prevotella copri*	30.39	10.78	12.14	24.20	6.35	0.029	0.877	0.130
*Lactobacillus kitasatonis*	10.39	4.94	3.90	1.07	3.95	0.213	0.812	0.376
*Lactobacillus mucosae*	7.46	5.32	3.59	2.86	2.33	0.482	0.570	0.420
*Pelomonas puraquae*	0.79	2.96	8.84	1.67	2.56	0.550	0.110	0.722
*Lactobacillus delbrueckii*	3.77	6.58	2.09	2.39	1.71	0.245	0.065	0.085
*Prevotella* sp.	1.55	3.66	3.28	4.87	1.89	0.435	0.888	0.651
*Prevotella stercorea*	5.25	0.96	1.49	4.54	1.19	0.011	0.747	0.033
*Streptococcus infantarius*	2.77	2.34	4.74	1.68	1.58	0.847	0.286	0.768
*Streptococcus alactolyticus*	3.66	1.90	3.88	2.60	0.99	0.179	0.132	0.590
*Propionibacterium acnes*	0.30	2.04	7.13	1.28	1.25	0.329	0.006	0.669
*Lactobacillus* sp.	2.37	4.24	1.58	1.84	1.28	0.294	0.136	0.178
*Helicobacter* sp.	3.60	0.72	1.61	1.66	1.26	0.113	0.622	0.603
*Corynebacterium glutamicum*	0.20	1.29	6.73	0.80	1.62	0.630	0.019	0.831
*Sphingomonas pseudosanguinis*	0.73	0.98	2.84	0.42	0.61	0.743	0.016	0.459
*Campylobacter upsaliensis*	0.77	0.20	1.73	0.35	0.72	0.558	0.119	0.877
*Lactobacillus ruminis*	1.63	0.36	0.71	0.25	0.64	0.168	0.703	0.903
*Roseburia faecis*	1.25	0.45	0.20	0.63	0.26	0.033	0.508	0.628
*Dialister succinatiphilus*	0.40	0.27	0.49	0.99	0.25	0.724	0.535	0.049
*Succinivibrio dextrinosolvens*	0.52	1.01	0.70	0.24	0.39	0.376	0.581	0.168
*Haemophilus felis*	0.06	0.92	0.05	1.27	0.63	0.334	0.332	0.694
Others	8.51	9.10	12.29	9.77	1.93	0.831	0.249	0.807

^a^Treatments: NC, no challenge/no supplement; PC, F18^+^ *E. coli* challenge/no supplement; ATB, F18^+^ *E. coli* challenge/bacitracin (30 g/t feed); and LPB, F18^+^ *E. coli* challenge/*Lactobacillus* postbiotics (2 kg/t feed; LBiotix, Adare Biome, Houdan, France)

**Fig. 1:**
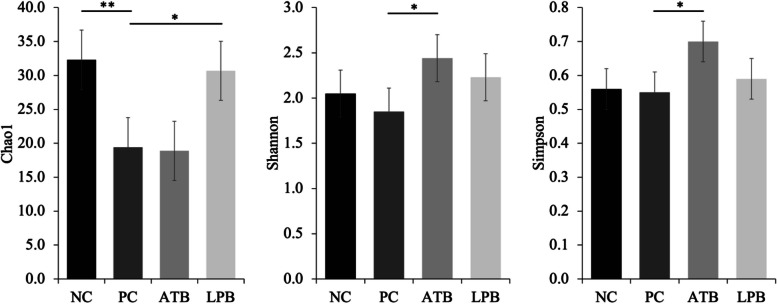
Alpha diversity (Chao1, Shannon, Simpson) of the jejunal mucosa-associated microbiota in pigs challenged with F18^+^ *Escherichia coli* (*E. coli*) and fed diets supplemented with bacitracin or *Lactobacillus* postbiotics. ^**^P < 0.05; ^*^0.05 ≤ *P* < 0.10

**Fig. 2:**
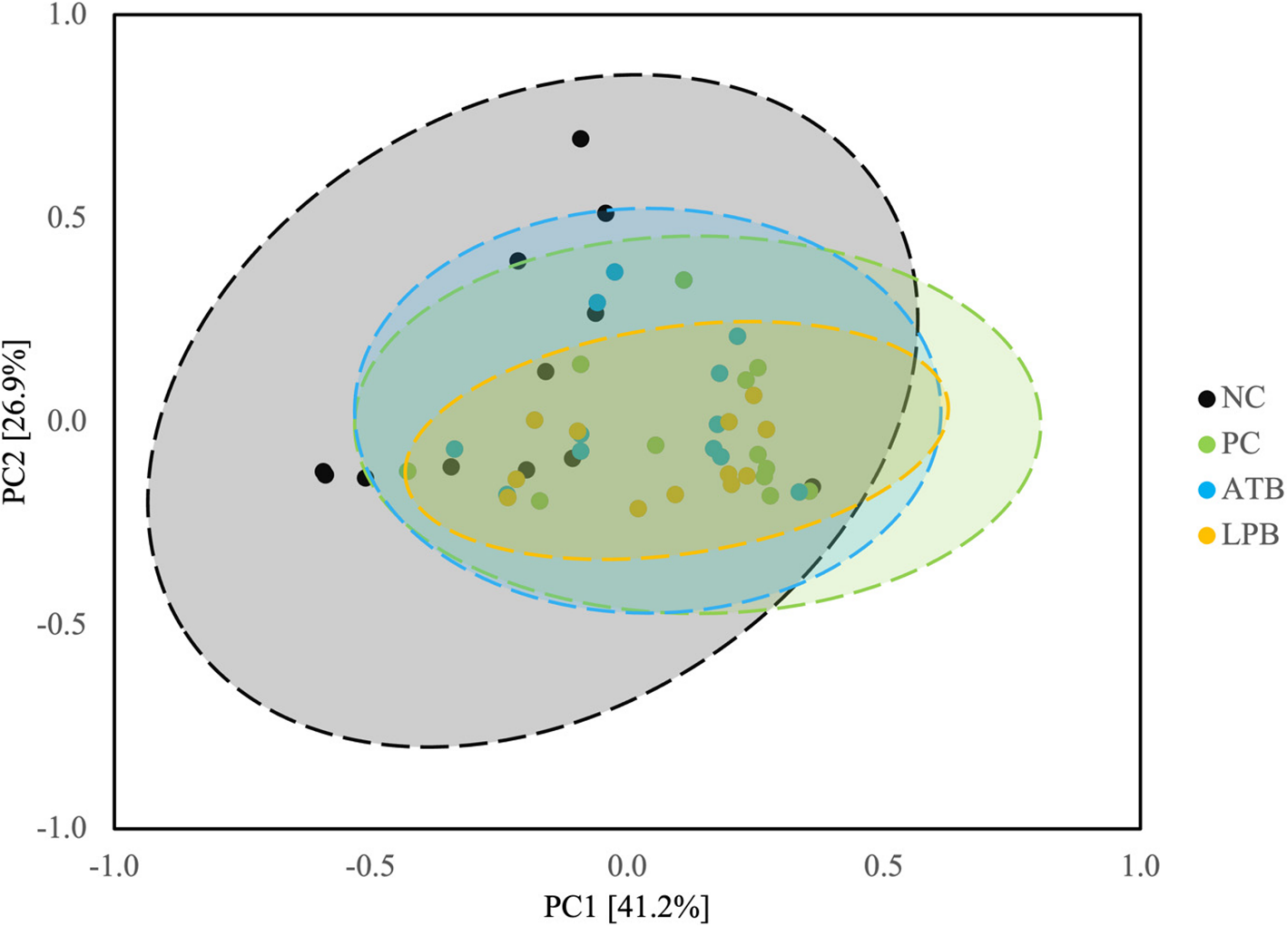
Principal component analysis (PCA) plot at genus level. The *X*-axis and *Y*-axis represent the principal component axes, with the percentages indicating the proportion of variation explained by each component. Points of different colors correspond to samples from different treatments, and the closer two points are, the more similar their species composition

The alpha diversity of mucosa-associated microbiota estimated with Chao1 in pigs on PC was lower (*P* < 0.05) than in pigs on NC (Fig. 1) and tended to be lower (*P* = 0.076) than in pigs on LPB. The alpha diversity of mucosa-associated microbiota estimated with Shannon in pigs on PC tended to be lower (*P* = 0.081) than in pigs on ATB. The alpha diversity of mucosa-associated microbiota estimated with Simpson in pigs on PC tended to be lower (*P* = 0.078) than in pigs on ATB. The microbial community was visualized using PCA based on Bray-Curtis distance, which confirmed PC changed (*P* < 0.05) microbiota composition in the jejunal mucosa of nursery pigs compared to NC (Fig. 2). The ATB and LPB did not affect beta diversity in pigs challenged with F18^+^ *E. coli*.

### Intestinal markers

**Fig. 3:**
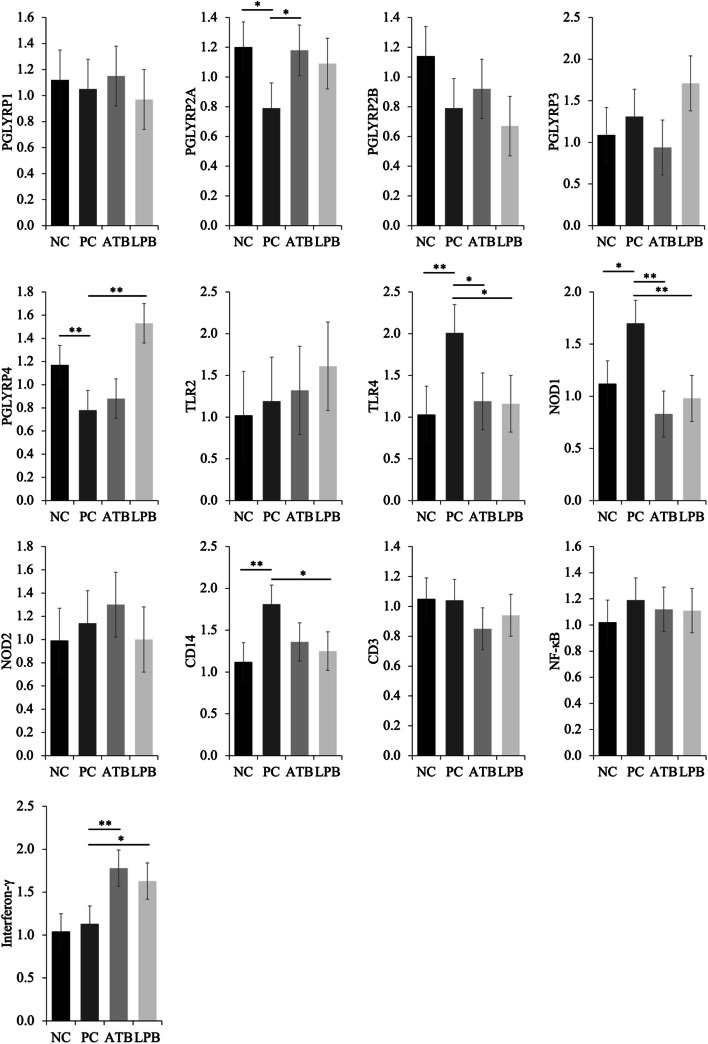
Relative gene expression of pattern recognition receptors in the jejunal mucosa of pigs challenged with F18^+^ *Escherichia coli* (*E. coli*) and fed diets supplemented with bacitracin or *Lactobacillus* postbiotics. PGLYRP, peptidoglycan recognition protein; TLR, toll-like receptor; NOD, nucleotide-binding oligomerization domain-containing protein; CD, cluster of differentiation; NF-κB, nuclear factor kappa B. ^**^P < 0.05; ^*^0.05 ≤ *P* < 0.10

The PC tended to reduce (*P* = 0.097) the relative expression of *PGLYRP2A* in the jejunum of nursery pigs when compared with NC, however, the ATB tended to increase (*P* = 0.074) *PGLYRP2A* when compared with PC (Fig. 3). The ATB tended to reduce (*P* = 0.099) the relative expression of *PGLYRP3* when compared with PC. The LPB increased (*P* < 0.05) the relative expression of *PGLYRP4* when compared with PC. The PC increased (*P* < 0.05) the relative expression of *TLR4* when compared with NC, however, the ATB tended to reduce (*P* = 0.085) *TLR4* when compared with PC. The PC increased (*P* < 0.05) the relative expression of *NOD1* when compared with NC, however, the ATB and the LPB reduced (*P* < 0.05) when compared with PC. The PC tended to increase (*P* = 0.079) the relative expression of *CD14* when compared with NC, however, the LPB tended to reduce (*P* = 0.091) when compared with PC. The ATB increased (*P* < 0.05) the relative expression of interferon-γ and the LPB tended to increase (*P* = 0.096) when compared with PC. The relative expressions of *PGLYRP1*, *PGLYRP2B*, *TLR2*, *NOD2*, *CD14*, *CD3*, and *NF-κB* were not affected by the treatments.

### Immune parameters and oxidative stress

The concentration of IL-8 in the jejunal mucosa of pigs was not affected by treatments (Table 6). The concentration of TNF-α in the jejunal mucosa of pigs fed PC tended to be higher (*P* = 0.084) than pigs fed NC and was higher *(P* < 0.05) than pigs fed ATB and LPB. The concentration of MDA in the jejunal mucosa of pigs fed PC was higher (*P* < 0.05) than pigs fed NC, ATB, and LPB, whereas the concentration of protein carbonyl was not affected by the treatments.

**Table 6:** Oxidative stress and immune parameters of pigs challenged with F18^+^ *Escherichia coli* (*E. coli*) and fed diets supplemented with bacitracin or *Lactobacillus* postbiotics

Item	Treatment^a^				SEM	*P*-value		
	NC	PC	ATB	LPB		NC vs. PC	PC vs. ATB	PC vs. LPB
IL-8, ng/mg	0.591	0.655	0.712	0.639	0.061	0.446	0.513	0.845
TNF-α, pg/mg	0.885	1.036	0.811	0.833	0.060	0.084	0.012	0.022
MDA, nmol/mg	0.301	0.564	0.305	0.301	0.076	0.010	0.013	0.021
Protein carbonyl, nmol/mg	0.985	1.087	1.040	0.999	0.088	0.402	0.699	0.466

^a^Treatments: NC, no challenge/no supplement; PC, F18^+^ *E. coli* challenge/no supplement; ATB, F18^+^ *E. coli* challenge/bacitracin (30 g/t feed); and LPB, F18^+^ *E. coli* challenge/*Lactobacillus* postbiotics (2 kg/t feed; LBiotix, Adare Biome, Houdan, France)

### Jejunal barrier function

**Fig. 4:**
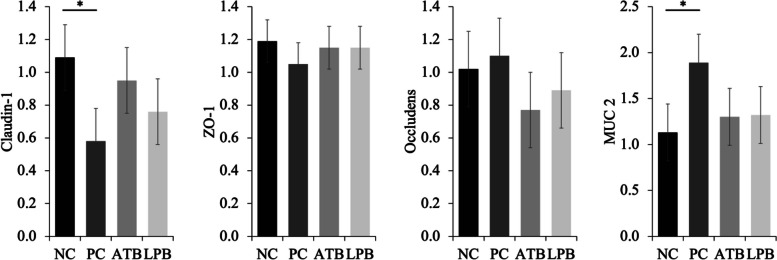
Relative gene expression of intestinal tight junction proteins and mucin in pigs challenged with F18^+^ *Escherichia coli* (*E. coli*) and fed diets supplemented with bacitracin or *Lactobacillus* postbiotics. ZO-1, zonula occluden-1; MUC2, mucin 2. ^**^P < 0.05; ^*^0.05 ≤ *P* < 0.10

The ATB tended to increase the relative expression of claudin-1 in the jejunum of pigs when compared with PC (Fig. 4). However, the relative expressions of *ZO-1*, occludens, and *MUC2* were not affected by the treatments.

### Jejunal morphology and crypt cell proliferation

**Fig. 5:**
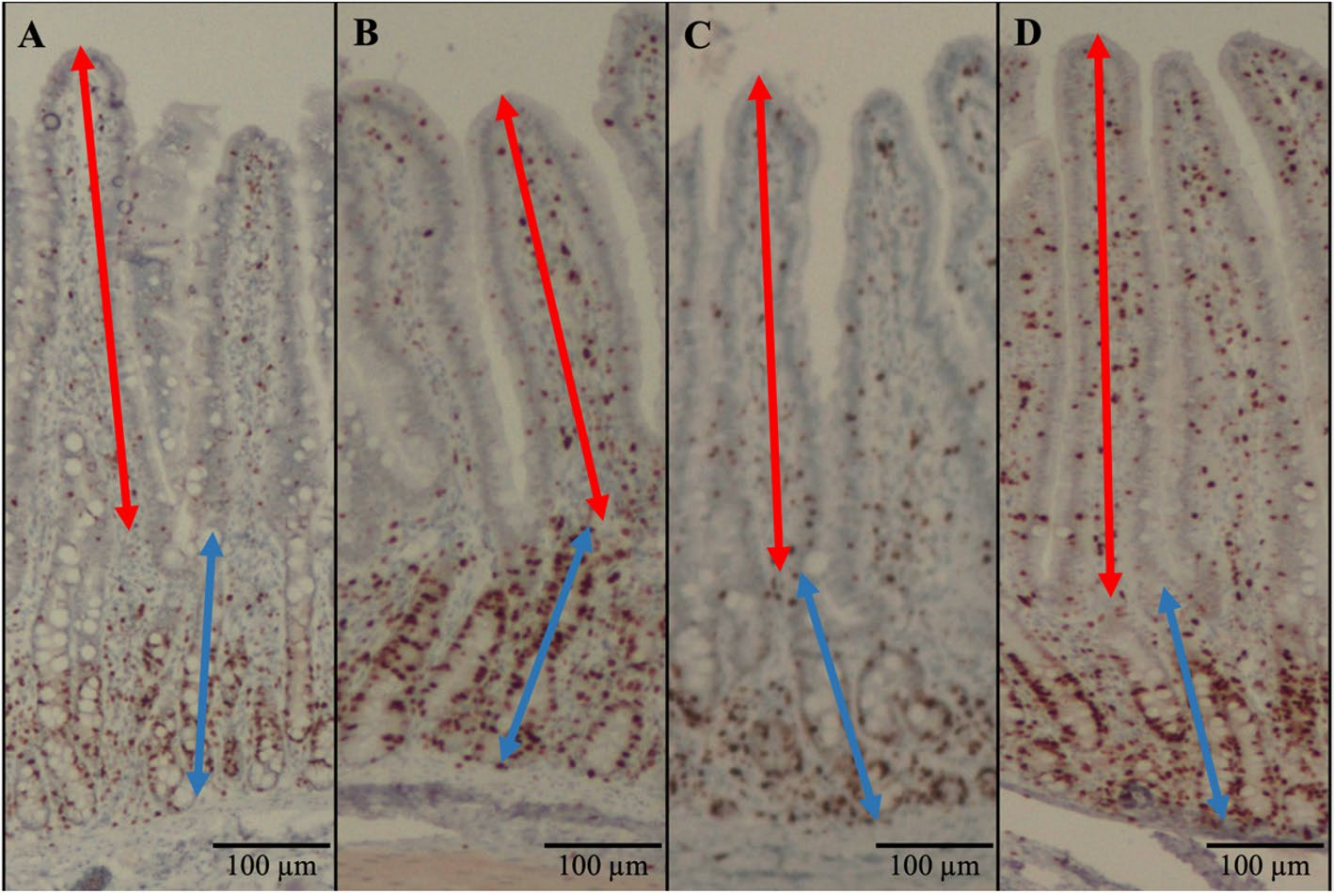
Representative images of the immunohistochemistry (Ki67) staining for the jejunal morphology. **A**–**D** were representative images of intestinal morphology from treatment NC, PC, ATB, and LPB; Ten images at 40× of well-oriented villi and their associated crypts were obtained to measure villus height (from the top to the base of villus, indicated by a red double arrow line) and crypt depth (from the base of villus to the bottom of the crypt, indicated by a blue double arrow line)

**Fig. 6:**
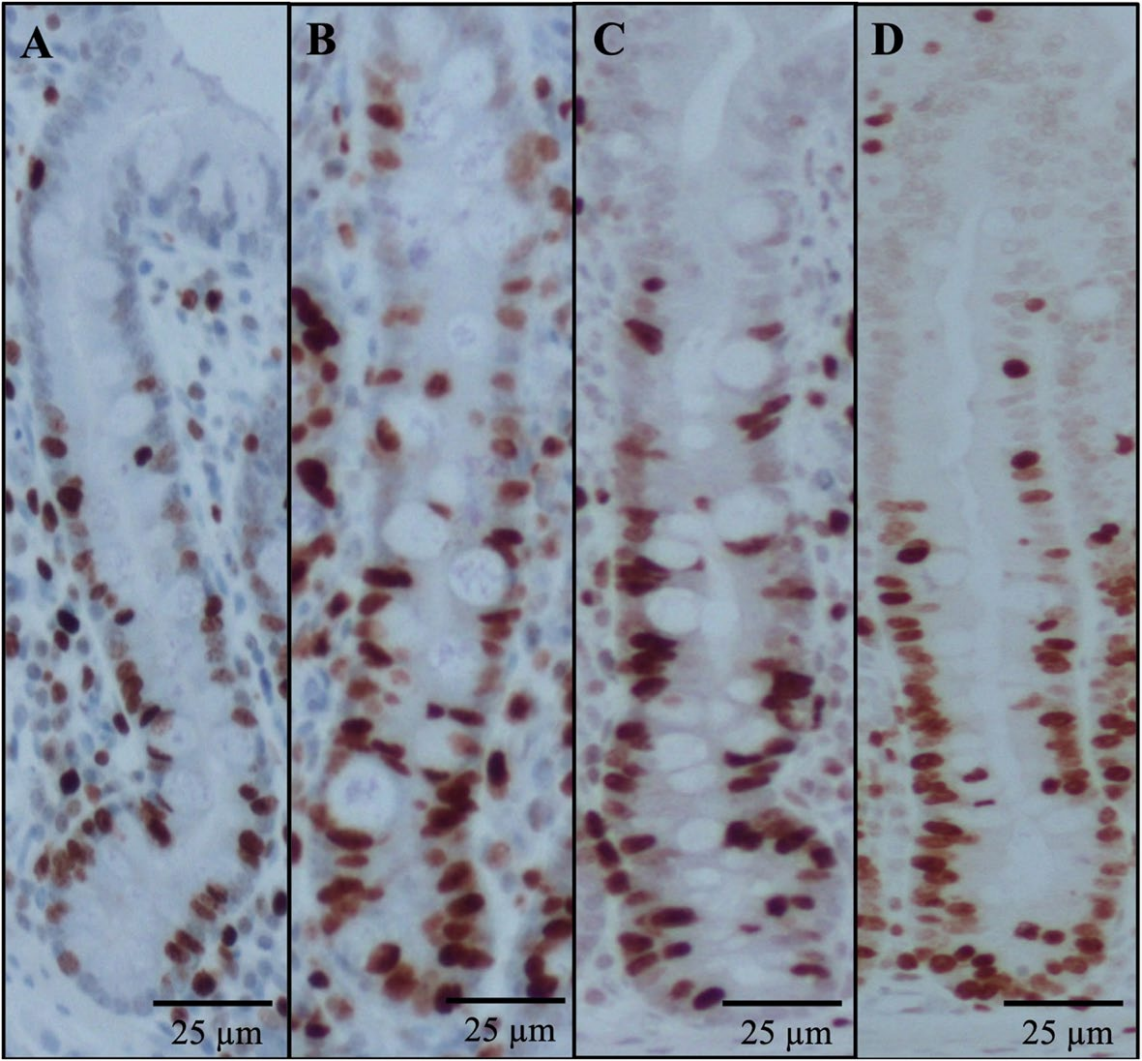
Representative images of the immunohistochemistry (Ki67) staining for the proliferation cells in crypt. **A**–**D** were representative images of intestinal morphology from treatment NC, PC, ATB, and LPB. Ten images at 100× of the crypts were obtained to measure the percentage of positive Ki67 staining cells (red)

The villus height, villus width, crypt depth, and VH:CD in the jejunum of pigs were not affected by treatments (Table 7; Fig. 5 and 6). The crypt cell proliferation rate in pigs fed PC was higher (*P* < 0.05) than in pigs fed NC. The LPB did not affect the crypt cell proliferation rate in pigs challenged with F18^+^ *E. coli*.

**Table 7:** Jejunal morphology and crypt cell proliferation in pigs challenged with F18^+^ *Escherichia coli* (*E. coli*) and fed diets supplemented with bacitracin or *Lactobacillus* postbiotics

Item	Treatment^a^				SEM	*P*-value		
	NC	PC	ATB	LPB		NC vs. PC	PC vs. ATB	PC vs. LPB
Villus height, µm	455	445	448	442	20	0.718	0.917	0.934
Villus width, µm	127	126	125	118	4	0.856	0.815	0.167
Crypt depth, µm	259	259	239	249	9	0.992	0.130	0.427
VH:CD^b^	1.76	1.72	1.91	1.78	0.13	0.707	0.111	0.556
Ki-67^+c^, %	17.6	21.9	22.4	20.7	3.0	0.028	0.783	0.551

^a^Treatments: NC, no challenge/no supplement; PC, F18^+^ *E. coli* challenge/no supplement; ATB, F18^+^ *E. coli* challenge/bacitracin (30 g/t feed); and LPB, F18^+^ *E. coli* challenge/*Lactobacillus* postbiotics (2 kg/t feed; LBiotix, Adare Biome, Houdan, France)

^b^Villus height to crypt depth ratio

^c^Ratio of Ki-67 positive cells to total cells in the crypt to indicate the crypt cell proliferation rate

### Fecal score

The fecal score of pigs was not affected by the treatments at d 7 (prior to F18^+^ *E. coli* challenge; pre-challenge), however, immediately after the challenge on d 7, pigs fed PC had higher (*P* < 0.05) fecal score than pigs fed NC (Table 8). At d 14 pigs fed LPB tended to have lower (*P* = 0.078) fecal scores than pigs fed PC, whereas the fecal score of pigs fed PC tended to be higher than pigs fed ATB at d 21 (*P* = 0.057) and 28 (*P* = 0.057).

**Table 8:** Fecal score of pigs challenged with F18^+^ *Escherichia coli* (*E. coli*) and fed diets supplemented with bacitracin or *Lactobacillus* postbiotics

Item	Treatment^a^				SEM	*P*-value		
	NC	PC	ATB	LPB		NC vs. PC	PC vs. ATB	PC vs. LPB
d 7 (pre-challenge)	3.50	3.17	3.42	3.34	0.17	0.155	0.284	0.473
d 7 (post-challenge)	3.36	4.11	3.61	4.19	0.27	0.048	0.183	0.823
d 14	3.41	3.33	3.08	2.99	0.13	0.655	0.183	0.078
d 21	3.02	3.10	2.85	3.19	0.11	0.520	0.057	0.520
d 28	2.93	3.10	2.85	3.02	0.09	0.237	0.079	0.552

^a^Treatments: NC, no challenge/no supplement; PC, F18^+^ *E. coli* challenge/no supplement; ATB, F18^+^ *E. coli* challenge/bacitracin (30 g/t feed); and LPB, F18^+^ *E. coli* challenge/*Lactobacillus* postbiotics (2 kg/t feed; LBiotix, Adare Biome, Houdan, France)

### Growth performance

The BW of pigs was not affected by the treatments during the experimental period (Table 9). The ADG of pigs was not affected by the treatments at the pre-challenge period (d 0 to 7). However, from d 7 to 14 (post-challenge) the ADG of pigs fed PC diet tended to be lower than pigs fed NC diet (*P* = 0.067), whereas, in overall, no differences were found between PC vs. ATB, or PC vs. LPB. Overall, the ADFI of pigs was not affected by the treatments. However, the ADFI of pigs fed LPB was lower (*P* < 0.05) than pigs fed PC at d 14 to 21 and tended to be lower (*P* = 0.088) at d 7 to 21. The G:F of pigs was not affected by the treatments at the pre-challenge period (d 0 to 7). However, from d 7 to 14 (post-challenge) the G:F of pigs fed PC diet tended to be lower than pigs fed NC diet (*P* = 0.054), whereas the G:F of pigs fed LPB tended to greater than pigs fed PC in overall (*P* = 0.069).

**Table 9:** Growth performance of pigs challenged with F18^+^ *Escherichia coli* (*E. coli*) and fed diets supplemented with bacitracin or *Lactobacillus* postbiotics

Item	Treatment^a^				SEM	*P*-value		
	NC	PC	ATB	LPB		NC vs. PC	PC vs. ATB	PC vs. LPB
BW, kg								
Initial	7.89	7.83	7.88	7.81	0.47	0.694	0.738	0.929
d 7	8.13	8.09	8.33	8.38	0.42	0.892	0.403	0.315
d 14	11.23	10.58	10.91	10.63	0.68	0.175	0.495	0.922
d 21	15.76	15.32	16.02	15.43	0.76	0.473	0.260	0.858
d 28	20.56	20.18	21.65	20.77	0.95	0.673	0.108	0.516
ADG, g/d								
d 0 to 7	34	38	65	82	23	0.892	0.361	0.133
d 7 to 14	440	352	364	318	48	0.067	0.799	0.457
d 14 to 21	647	677	730	686	42	0.622	0.369	0.877
d 21 to 28	685	694	804	762	48	0.894	0.109	0.316
d 7 to 28	589	572	631	586	30	0.694	0.162	0.733
d 0 to 28	452	441	492	463	23	0.721	0.113	0.494
ADFI, g/d								
d 0 to 7	173	153	154	164	21	0.486	0.981	0.703
d 7 to 14	708	659	711	614	49	0.389	0.361	0.433
d 14 to 21	908	947	1003	795	61	0.576	0.428	0.035
d 21 to 28	1097	1102	1151	1072	43	0.927	0.410	0.613
d 7 to 28	846	844	894	770	49	0.969	0.312	0.137
d 0 to 28	709	702	742	650	36	0.833	0.349	0.219
G:F								
d 0 to 7	0.21	0.25	0.43	0.50	0.13	0.835	0.324	0.164
d 7 to 14	0.64	0.53	0.51	0.51	0.05	0.054	0.718	0.704
d 14 to 21	0.72	0.75	0.74	0.88	0.08	0.763	0.877	0.224
d 21 to 28	0.63	0.64	0.70	0.71	0.04	0.934	0.276	0.162
d 21 to 28	0.70	0.69	0.70	0.76	0.03	0.765	0.745	0.130
d 0 to 28	0.65	0.64	0.66	0.72	0.03	0.810	0.523	0.069

^a^Treatments: NC, no challenge/no supplement; PC, F18^+^ *E. coli* challenge/no supplement; ATB, F18^+^ *E. coli* challenge/bacitracin (30 g/t feed); and LPB, F18^+^ *E. coli* challenge/*Lactobacillus* postbiotics (2 kg/t feed; LBiotix, Adare Biome, Houdan, France)

## Discussion

The gastrointestinal tract is a complex ecosystem where the composition of the microbiota plays a pivotal role in shaping the immune responses of the host. The primary objective of this study was to provide a comprehensive understanding of the impact of an F18^+^ *E. coli* infection on the mucosa-associated microbiota and the immune responses in the jejunum of nursery pigs. The F18^+^ *E. coli* primarily targets the mucosa of the jejunum causing inflammation and mucosal damages in nursery pigs [7, 8, 27, 31]. The mucosa-associated microbiota plays an important role on the intestinal immune system [3, 20, 21]. On the other hand, however, the host immune system can also, modulate the microbiome within the intestine [21, 24]. Additionally, this study investigated the potential efficacy of dietary bacitracin and *Lactobacillus* postbiotics in mitigating or preventing the detrimental consequences induced by the infection. Understanding these interactions can provide valuable insights into strategies for promoting intestinal health and enhancing immune competence in young animals.

In the current study, pigs challenged with F18^+^ *E. coli* had modulated mucosa-associated microbiota by increasing the abundance of Proteobacteria, whereas reducing Firmicutes and Bacteroidetes. Previous studies have reported that *E. coli* infection can disrupt the intestinal microbiota in pigs [12, 27, 41–43] and humans [44, 45]. Duarte et al. [31] challenged nursery pigs with F18^+^ *E. coli* and demonstrated that F18^+^ *E. coli* counts were increased in the jejunal mucosa and fecal samples showing effectiveness of F18^+^ *E. coli* challenge. The changes in the composition of the mucosa-associated microbiota have been attributed to the disturbance of the fluidity of the intestinal electrolytes [5], characterized by the increased fecal score in this study. Enterotoxigenic *E. coli* secretes enterotoxins, including STa and STb, that bind to Guanylyl Cyclase C (GC-C) and sulfatide, respectively, in the enterocytes [46–48]. The interaction between enterotoxins and receptors on the intestinal mucosa triggers the production of cellular cyclic adenosine monophosphate (cAMP), initiating reactions that disturb the balance of electrolyte fluid in the intestine, consequently resulting in watery diarrhea [7, 49–51].

The disturbed electrolyte fluidity in the intestine creates favorable conditions for the proliferation of bacteria from the phylum Proteobacteria, including *Helicobacter* spp. [5, 29]. The high abundance of *Helicobacter mastomyrinus* observed in the jejunal mucosa in this study is in line with the findings reported by Duarte and Kim [27]. *Helicobacter* spp. are Gram-negative, aerobic microorganisms exhibiting notable motility and adherence properties [52, 53] which contribute to their advantageous establishment within the mucosal lining of the small intestine of challenged pigs. The overgrowth of *Helicobacter* spp., consequently resulted in reduced microbial diversity. Previous studies reported that *Helicobacter* is positively correlated with pro-inflammatory cytokines in the jejunal mucosa [12, 27]. Moreover, the observed increase in inflammatory microbiota and reduced microbial diversity in response to the *E. coli* challenge aligns with previous research that demonstrated the disruption of intestinal microbiota during pathogenic infections leading to detrimental effects on intestinal health markers [54, 55].

The use of dietary bacitracin increased the abundance of Propionibacteriaceae, Comamonadaceae, Corynebacteriaceae, and Sphingomonadaceae increasing microbial diversity. Bacitracin is a peptide produced by *Bacillus subtilis* that interferes with the synthesis of peptidoglycan in the growing bacterial cell [56, 57]. Recent studies have demonstrated that bacitracin can alleviate the deleterious effects of F18^+^ *E. coli* in nursery pigs under experimental conditions [12, 31]. These results are possibly due to the presence of peptidoglycan in the cell wall of Gram-negative bacteria, although at a lower level when compared with Gram-positive bacteria [58].

Similar to bacitracin, the postbiotics modulated the mucosa-associated microbiota by increasing the abundance of *Prevotella stercorea*, and *Dialister succinatiphilus* which also resulted in increased microbial diversity. *Lactobacillus* postbiotics have long been used to promote intestinal health, modulating the intestinal microbiota and preventing or mitigating the effects of pathogenic infection [12, 17, 59–62]. Postbiotics have been defined as a formulation of non-living microorganisms and their constituents that provide health benefits to the target host [14]. The postbiotics used in the current study contains heat-stabilized *Lactobacillus fermentum* and *L. delbrueckii*, their metabolites produced during fermentation, and bacterial debris from the spent culture. It can be speculated that these components can modulate the microbiota by increasing the competitiveness for receptors within the intestine, by directly affecting the growth of commensal bacteria, or by triggering immune responses that prevent the adherence of pathogens [63]. One component in the postbiotics used in the present study is peptidoglycan, present in the cell wall of *Lactobacillus* spp. Peptidoglycans can induce the production of immunoglobulins through the activation of TLR2 [22, 23]. The immunoglobulins secreted into the intestinal lumen can coat bacterial cell walls, limiting their attachment and preventing translocation to the mucosal epithelium [64–66].

The mucosa-associated microbiota is one of the first lines of defense against opportunistic pathogens [67, 68]. Previous studies have demonstrated that the mucosa-associated microbiota plays a significant role in shaping the immune function of the intestinal system [25–27, 69]. It is noteworthy that many of the genes linked to the mucosa-associated microbiota are associated with immune responses, indicating that the immune system, on the other hand, actively influences the composition of the intestinal microbiota, fostering the development of beneficial microbial communities [24, 70, 71]. Under homeostasis status, commensal bacteria, including those from the phylum Proteobacteria, can trigger the immune system and help protect the barrier integrity of the intestine [3]. However, during stressful events including weaning, or under challenging conditions, the balance of the microbiota can be disturbed, increasing the chances of opportunistic pathogens overgrowth and leading to intestinal inflammation or enteric diseases [5, 27].

The intestinal microbiota and their metabolites are sensed by PRR in epithelial cells, including enterocytes, dendritic cells, and microfold cells triggering immune responses, consequently affecting the composition of intestinal microbiota [64, 72–74]. In this study, pigs challenged with F18^+^ *E. coli* had increased gene expression of *TLR4*, *CD14*, and *NOD1*, suggesting an active host response to microbial endotoxins. Toll-like receptor 4 and CD14 are PRR expressed in epithelial cells that recognize the endotoxins produced by Gram-negative bacteria, including LPS, facilitating the production of NF-κB, TNF-α, and IL-8 [75, 76]. In addition, NOD1, a cytosolic receptor, has been shown a synergistic interaction with TLR4 playing a central role in the recognition of Gram-negative bacteria and the activation of immune responses [77, 78]. Collectively, the upregulation of PRR genes in response to the F18^+^ *E. coli* challenge suggests an activated immune response triggering a pro-inflammatory cascade. These genes are crucial components of the innate immune system, which plays a pivotal role in recognizing and responding to pathogens. The subsequent trend of increase in the concentration of TNF-α in the jejunal mucosa associated with pro-inflammatory responses further confirms the efforts of the host to combat the pathogenic challenge.

Interestingly, the bacitracin showed a trend to upregulate the gene expression of *PGLYRP2A*, interferon-γ, and downregulated *TLR4* and *NOD1*. These results can be related to the modulation of the intestinal microbiota toward a more diverse and balanced composition. The postbiotics enhanced the immunocompetence of nursery pigs by increasing the expression of interferon-γ and *PGLYRP4*, reducing the expression of PRR genes including *NOD1* and *CD14*, which may indicate a reduction of the pathogen invasion. The gene *PGLYRP4* is mainly expressed in epithelial tissues and is been associated with changes on microbiota due to their bactericidal properties against Gram-negative and Gram-positive bacteria [79–81]. Interferon-γ is a vital immunoregulatory cytokine that plays a central role in combating bacterial infections [82, 83]. The increased expression of interferon-γ, *PGLYRP2A*, and *PGLYRP4* following bacitracin and postbiotic treatments indicates an enhancement of immune responses, whereas the reduction in the expression of PRR genes including *NOD1* and *CD14*, implies higher protection against pathogen invasion. Duarte et al. [31]. reported that the bacitracin enhanced intestinal health of nursery pigs by reducing the F18^+^ *E. coli* population and the relative abundance of *Helicobacter* spp. in the jejunal mucosa. *Lactobacillus* spp. has been associated with reduced inflammation and epithelial damage [84, 85]. The heightened inflammatory state in the challenged pigs led to an increase in oxidative damage in the jejunum. The inflammation caused by the *E. coli* infection can cause an unbalance in the antioxidant capacity by over-producing reactive oxygen species (ROS), resulting in oxidative damage in the intestinal mucosa [12, 29, 86]. In the current study, the concentration of MDA, a product of lipid oxidation, was increased in challenged pigs. Furthermore, an increased abundance of Proteobacteria has been associated with increased oxidative damage in the jejunal epithelium [12, 27, 29, 36]. Conversely, bacitracin and postbiotics reduced the concentration of MDA in the jejunal mucosa, possibly due to the reduction of inflammation.

Proteobacteria, including *Helicobacter* spp., are known to degrade the intestinal mucus layer [87, 88] further increasing inflammation and oxidative damage by reducing the physical protection from the intestine [27, 89, 90]. The reduced mucus layer in combination with the increased inflammatory response and the oxidative damage can increase the need for increased cell proliferation to promote epithelial repair. In this study, increased *MUC2* gene expression in the challenged group was observed, which indicates that the challenged pigs attempted to repair the damaged mucus layer. The cell proliferation rate in the crypt, reported as Ki67^+^, was increased in pigs challenged with F18^+^ *E. coli*. This result agrees with previous reports [12, 37] that reported increased cell proliferation in the jejunum of pigs challenged with F18^+^ *E. coli*. However, other parameters of intestinal morphology did not differ among treatments. In this study, crypt depth was not influenced by the treatment regardless of increased percentage of cells newly proliferating. Crypt cell proliferation was expressed by the percentage of proliferating cells, and it does not indicate the number of proliferating cells. Crypt depth could be extended if the number of proliferating cells increases whereas changes of the percentage of proliferating cells may not directly affect the crypt depth.

The growth performance reported in this study was not affected by the treatments, possibly due to the mild diarrhea caused by the F18^+^ *E. coli* challenge. Furthermore, it is important to mention that the results reported in the current study are from 21 d after the inoculation, indicating a long-lasting effect of F18^+^ *E. coli* infection on the mucosa-associated microbiota, as previously reported [27, 29]. The F18^+^ *E. coli* challenge employed is representative of a common pathogenic threat faced by nursery pigs around the world. Therefore, the findings presented in this study shed light on the significant impact of dietary interventions on mucosa-associated microbiota composition, immune responses through the modulation of PRR, and oxidative damage in nursery pigs challenged with F18^+^ *E. coli*. Furthermore, the understanding of the microbial and immunological responses in nursery pigs when exposed to F18^+^ *E. coli* infection suggests practical approaches for managing and preventing the deleterious effects of infections. The utilization of dietary *Lactobacillus* postbiotics emerges as a promising avenue for preventing enteric infection.

## Conclusion

The F18^+^ *E. coli* challenge increased harmful bacteria associated with the jejunal mucosa, upregulating the expression of pathogen recognition genes, including *TLR4*, *CD14*, and *NOD1*, increasing the production of cytokines associated with pro-inflammatory response. Bacitracin increased the abundance of beneficial bacteria showing a trend towards increasing the intestinal barrier function, possibly by reducing the expression of genes associated with pathogen recognition. *Lactobacillus* postbiotics enhanced the immunocompetence of nursery pigs by increasing the expression of interferon-γ and *PGLYRP4*, and by reducing the expression of genes associated with pathogen recognition (*TLR4*, *NOD1*, and *CD14*), which indicates reduced pathogen invasions.

## Data Availability

All data generated or analyzed during this study are available from the corresponding author upon reasonable request.

## Abbreviations

CD: Cluster of differentiation
*E*. *coli*: *Escherichia coli*
GC-C: Guanylyl cyclase C
IL-8: Interleukin 8
MDA: Malondialdehyde
NF-κB: Nuclear factor kappa B
NOD: Nucleotide-binding oligomerization domain-containing protein
OTU: Operational taxonomic unit
PRR: Pattern recognition receptors
PGLYRP: Peptidoglycan recognition protein
TLR: Toll-like receptor
TNF-α: Tumor necrosis alpha
